# Utility of an orthopaedic trauma registry in Ghana

**DOI:** 10.4314/gmj.v55i3.6

**Published:** 2021-09

**Authors:** Elissa K Butler, Dominic Konadu-Yeboah, Peter Konadu, Dominic Awariyah, Charles N Mock

**Affiliations:** 1 Harborview Injury Prevention & Research Center, University of Washington, 325 9th Ave Box 359960, Seattle, WA, USA; 2 Kwame Nkrumah University of Science and Technology and Department of Surgery (Orthopaedics and Trauma), Komfo Anokye Teaching Hospital, P.O. Box 1934, Kumasi, Ghana

**Keywords:** orthopaedic injury, trauma registry, Ghana, low- and middle-income country, data quality

## Abstract

In most low- and middle-income countries, trauma registries are uncommon. Although institutional registries for all trauma patients are ideal, it can be more practical to institute departmental registries for specific subsets of patients. Komfo Anokye Teaching Hospital (KATH) has started a locally developed, self-funded orthopaedic trauma registry. We describe methods and experiences for data collection and examine patient and injury characteristics, data quality, and the utility of the registry. Of 961 individuals in the registry, 67.9% were males, and the median age was 40 years. Motor vehicle collision (23.3%) was the most frequent mechanism of injury. Lower extremity fractures were the most common injury (60.6%), and 43.9% of injuries were managed operatively. Data quality was reasonable with missingness under 10% for 13 of 14 key variables, with inconsistencies of dates of injury, admission, treatment, and discharge in 9.1% of cases. However, the type of operation was missing for 73.2% of operative cases. Despite these limitations, the registry has been used for quality improvement and to successfully advocate for resources to improve trauma care. The registry has been improved by adding more detailed outcome variables, creating a standardised codebook of categorical variables, and adding more fields to allow for multiple injuries. In conclusion, it is practical and sustainable to institute a locally developed, self-funded orthopaedic trauma registry in Ghana that provides data with reasonable quality. Such a registry can be used to advocate for more resources to care for injured patients adequately and for quality improvement.

**Funding:**

None declared

## Introduction

Injury is a significant cause of death and disability worldwide.^1^ Improving the organisation and planning for trauma care can affordably and sustainably lower trauma mortality rates in countries at any economic level^2^ A key tool for accomplishing this is a trauma registry, which is defined as “a disease-specific collection composed of a file of uniform data elements that describe the injury event, demographics, pre-hospital information, diagnosis, care, outcomes, and cost of treatment for injured patients”.^3^ Trauma registries can be used in quality improvement initiatives, trauma severity scoring, injury prevention projects, planning resource allocation, and tracking performance in trauma care over time.^4^ In high-income countries, the implementation of trauma registries has led to the reorganisation of trauma care into regional systems and has been associated with decreased mortality due to injuries.^5^–^7^

In low- and middle-income countries (LMICs), trauma registries are rare.^8^ In a scoping review of publications on trauma registries, O'Reilly *et al.* found that only 4% of publications on registries referred to countries in the low and medium group of the United Nations Development Index.^9^ Several publications have generally discussed trauma registries in LMICs. However, these are often short-term research reports rather than sustainable, institutional registries implemented for qualityimprovement purposes 8. Data quality issues, limited funding, technology, human resources, infrastructure, and administrative and organisational difficulties are significant barriers to creating sustainable trauma registries, particularly in LMICs.^10^ Although sustainable, institutional registries for all trauma patients are ideal. In some cases, it can be more feasible to institute departmental registries for specific subsets of patients.

In high-income countries, several groups have created orthopaedic-specific trauma registries. The main objectives of these registries are to include all patients with musculoskeletal injury (not just individuals with severe multi-system injury) and collect data on specific orthopaedic injury patterns, treatments, and outcomes.^11^ In 2003, the Victorian Orthopaedic Trauma Outcomes Registry (VOTOR) was created to include patients with orthopaedic injury admitted to two Level 1 trauma centres in Victoria, Australia.^12^ Since that time, VOTOR has been used to assess the epidemiology, treatment, and post-injury outcomes of specific injuries, including traumatic knee dislocations, proximal humerus fractures, Achilles tendon injuries, distal femur fractures, and medial clavicle fractures.^13^–^17^ In the United States, the Military Orthopaedic Trauma Registry (MOTR) was created in 2013, which collects information on a specific limb, fracture classifications, associated injuries per limb, number and timing of debridements, antibiotics, and implant types.^18^,^19^ The German Society for Orthopaedics and Trauma created a network of 14 orthopaedic registries that have been used for outcomes research, quality assurance projects, and the development of treatment recommendations with high levels of evidence.^20^,^21^

In LMICS, a few orthopaedic trauma registries have been created. The Section of Orthopaedics at Aga Khan University Hospital in Pakistan created an orthopaedic trauma database in 2015 that includes all individuals with traumatic upper or lower extremity fractures. They have used this registry to inform priority setting and planning, preventive strategies and management protocols, and ongoing data collection.^22^,^23^ A pilot orthopaedic trauma registry in a Ugandan district hospital was created and demonstrated delayed access to care in specific groups, including subsistence farmers, motorcycle taxi drivers, and preschool children. However, this appears to be a one-time report and data collection is not ongoing.^24^ Finally, a regional pelvic trauma database has been created in Hunan Province, China, that includes patient and injury characteristics, fracture management, complications, and outcomes. This registry is electronic and allows for user-friendly queries for data analysis.^25^

The Department of Trauma and Orthopaedic Surgery at Komfo Anokye Teaching Hospital (KATH) in Kumasi, Ghana, has started an orthopaedic trauma registry to better document and organise the care it provides a department. The objective of the current article is to examine the patient and injury characteristics of individuals included in the database, experiences in data collection and management, the quality of the data, and the utility of the registry.

By so doing, we hoped to demonstrate lessons learned that would be useful for other institutions contemplating starting an orthopaedic trauma registry.

## Methods

### Setting

Komfo Anokye Teaching Hospital (KATH) is a 1,500-bed hospital with a catchment population of approximately 10 million people and serves as the main referral hospital for the middle third of Ghana. It is a training centre for medical students at the Kwame Nkrumah University of Science and Technology and a centre for post-graduate training in several specialties, including orthopaedics.

### Patient selection and data collection

Data collection for the KATH orthopaedic trauma registry began on January 1 2017, and is ongoing. All adults (≥18 years) admitted to KATH with an orthopaedic injury were approached for inclusion in the orthopaedic trauma registry. Inclusion criteria include orthopaedic trauma (fractures and dislocations) in patients 18 years and above who presented to KATH. Exclusion criteria include non-orthopaedic trauma such as lacerations, head injuries, maxillofacial injuries, abdominal trauma. For this particular article, we restricted the analysis to patients admitted from January 1, 2017, to October 31, 2018.

After obtaining written informed consent, three trained research assistants prospectively extracted relevant data from patients' medical records and entered data into a Microsoft Visual Basic database (Redmond, Washington, USA) on password-protected computers. Initial variables included in the orthopaedic trauma registry were age, sex, occupation, history of drug or alcohol use, insurance status, date of injury, mechanism of injury, road safety equipment used (if applicable), method of transport to the hospital, whether the patient was referred from another facility, date and time of admission, orthopaedic diagnoses, date of initial treatment, operative versus non-operative treatment, treatment details, and date of discharge. If the information on diagnosis or management was unclear, the research assistants sought clarification from treating surgeons and nurses. Research assistants talked with patients or their relatives to obtain data on socioeconomic status. Data gathering began at the time of patient arrival at the hospital and continued throughout the hospital stay.

The orthopaedic trauma registry is run through minimal institutional funding. The research assistants perform various other activities and undertake data gathering and entry for this registry as an additional duty.

### Research assistants' qualifications and methods for assuring data accuracy

The research assistants all have first degrees (BSc). In addition, they have received training on research methodology, use of REDCap software, orthopaedic trauma data capture, fracture classification, and assessment of patient socioeconomic status. The Ghana College of Physicians and Surgeons' training was run in conjunction with the AO Alliance, an international orthopaedic trauma organisation.

Accuracy of data collection was assured by close supervision of the research assistants and by cross-checking of data in the medical records. Data captured by research assistants were reviewed weekly and in real-time by the Head of Department, other specialist orthopaedic surgeons, and senior residents in orthopaedics. This was done several times. Reviews were first done at the daily post-emergency duty ward rounds at 9 AM each day. Research assistants joined the rounds with the surgeons. Research assistants verified the diagnoses with the surgeons before entering the data on tablets which they carried on them during the ward rounds. During this time, data from the medical records were also crosschecked against the data entered by the research assistants for approximately 80% of cases. Such real-time reviews were supplemented by a weekly meeting with the Head of Department.

### Experiences in data collection and management

No patients who were approached to be included in the orthopaedic trauma registry declined participation. Data were primarily available in the medical record, but RAs occasionally had to get clarification from doctors or nurses taking care of the patients. There was sometimes inadequate documentation in referral notes from referring facilities and Emergency Department (ED) documentation. Additionally, there were occasionally missing medical records. Data abstraction required approximately 25 minutes per patient, based on estimates from the RAs. The research assistants were given a duty roster to ensure that data capture continued on weekends and public holidays. There was no difficulty in capturing all patients with three research assistants and active supervision by the lead surgeon. One large barrier to complete data capture was following patients after discharge. Many patients were discharged from the ED with splinting with instructions to follow up in the outpatient clinic to schedule definitive surgical management. Some patients did not return to the outpatient clinic, so the orthopaedic trauma registry does not include further management. However, even if patients did return as outpatients, the research assistants often had difficulty arranging to meet them during their clinic visits to complete data collection.

### Ethical considerations

Both the orthopaedic registry itself and the use of the data for this article were approved by the KATH Institutional Review Board (KATH IRB/AP/088/20). In addition, analysis of de-identified data for this article was considered exempt by the University of Washington Institutional Review Board.

### Data analysis

For this article, data were exported from the orthopaedic trauma registry and imported into STATA/SE version 14 (StataCorp LP, College Stations, Texas, USA) for data analysis. Patient and injury characteristics and management were described with counts and percentages for categorical variables and median and interquartile range (IQR) for continuous variables. During data cleaning, areas for improvement in data management were assessed. To assess data quality, internal inconsistencies were evaluated. Days from injury to admission, days from admission to treatment, and hospital length of stay were calculated. The frequency and per cent of implausible values (defined as <0 or ≥90 days) were determined. The level of missingness of 14 key variables that applied to all patients was investigated (age, sex, mechanism of injury, referral status, injury location, injury type, fracture severity, general treatment, detailed treatment, date of injury, date of admission, date of treatment, and date of discharge). We assessed two key performance indicators: proportion of femur fractures that underwent fixation and proportion of open fractures that underwent operative management.^26^

## Results

A total of 961 individuals with orthopaedic injury were included in the orthopaedic trauma registry from January 1 2017 to October 31 2018. The median age was 40 years (IQR: 29–56), and 67.9% were males (Table 1). Motor vehicle collisions (23.3%) and motorcycle collisions (20.1%) were the most frequent mechanisms of injury. Most patients (65.3%) arrived via public transport, and only 10.6% of patients arrived via ambulance. Almost half (48.2%) of patients were initially seen at another facility and referred to KATH to further manage their injuries.

**Table 1:** Patient characteristics of 961 individuals in the KATH orthopaedic trauma registry, Kumasi, Ghana, 2017–2018

Characteristic	Registry Cohort n(%)
**Age, median (IQR), y**	40 (29–56)
**Male, No. (%)**	645 (67.9)
**Mechanism, No. (%)**	
**Motor vehicle collision**	218 (23.3)
**Motorcycle collision**	188 (20.1)
**Fall from standing**	175 (18.7)
**Fall from height**	147 (15.7)
**Pedestrian**	105 (11.2)
**Struck by or against**	68 (7.3)
**Firearm**	10 (1.1)
**Cut or stab**	9 (1.0)
**Other blunt**	17 (1.8)
**Transport type, No. (%)**	
**Public transport**	624 (65.3)
**Private vehicle**	230 (24.1)
**Ambulance**	101 (10.6)
**Referred from another facility, No. (%)**	444 (48.2)
**State insured, No. (%)**	544 (56.6)

There was a total of 966 orthopaedic injuries among 946 patients with recorded diagnoses (Table 2). Fractures of the tibia and/or fibula were the most common injuries (41.4%), followed by femur fractures (19.2%). Of fractures with known severity, 25.6% were open fractures (220/859). Over half of injuries were managed nonoperatively (55.0%). Of patients managed nonoperatively, 74.1% were managed with splint or plaster. Of patients managed operatively, the type of operation was not documented for 73.2% of cases.

**Table 2:** Injury characteristics of 946 individuals with 966 orthopaedic injuries in the KATH orthopaedic trauma registry, Kumasi, Ghana, 2017–2018

Injury characteristic	Orthopaedic injuries n(%)
**Injury location, No. (%)**	
**Tibia/Fibula**	400 (41.4)
**Femur**	185 (19.2)
**Radius/Ulna**	120 (12.4)
**Humerus**	101 (10.5)
**Clavicle**	39 (4.0)
**Shoulder**	23 (2.4)
**Pelvis**	20 (2.1)
**Foot**	29 (3.0)
**Patella**	19 (2.0)
Other*	30 (3.1)
**Injury type, No. (%)**	
**Fracture**	929 (96.2)
**Dislocation**	35 (3.6)
**Laceration**	1 (0.1)
**Traumatic amputation**	1 (0.1)
**Fracture Severity, No (%)**	
**Closed**	639 (68.8)
**Open**	220 (23.7)
**Unknown**	70 (7.5)
**General management, No. (%)**	
**Operative**	414 (43.1)
**Non-operative**	529 (55.0)
**Unknown**	18 (1.9)
**Detailed operative management, No. (%)**	
**External fixation**	10 (1.9)
**Internal fixation**	13 (2.5)
**Arthroplasty**	4 (0.8)
**Unknown**	387 (73.2)
**Detailed non-operative management, No. (%)**	
**Splint or plaster**	392 (74.1)
**Traction**	71 (13.4)
**Sling or collar**	55 (10.4)
**Pain management**	7 (1.3)
**Bedrest**	2 (0.4)

### Data quality and missing data

There were several main findings regarding data quality. Six of the 14 key variables assessed for quality in this article were entered as free text in the original database: mechanism of injury, injury location, injury type, fracture severity, general treatment, and detailed treatment. There were no standardised methods for categorising these variables. In addition, there was only one field to document all diagnoses for each patient, so it was occasionally unclear when patients had multiple orthopaedic injuries. Also, there was only one field for all treatments for each patient. For patients with multiple injuries, it was sometimes unclear which treatment pertained to which injury.

There were some inconsistencies in the data, particularly regarding the date of injury, date of admission, treatment date, and discharge date. There were 87 patients (9.1%) with an implausible calculated period (injury to admission, admission to treatment, or length of stay) (Table 3). Of patients with implausible dates, 23 (26.4%) had an obvious typographical error in one of the documented years (e.g. “2007” instead of “2017); however, the remaining patients had accurate years and inaccurate month or day of the month documented, resulting in implausible values for the calculations. An additional inconsistency observed in the data was that 31.7% of patients had “operative” documented for general management of their injury. Still, the detailed management included only non-operative treatment such as “splint” or “plaster.” For this article, we categorised a patient with any documentation of an operation (even if the type of operation was not specified) as having operative management. Based on the orthopaedic trauma registry alone, it was unclear if these patients received operative management for which the details were not recorded, or they were inaccurately documented as having operative management.

**Table 3:** Implausible time period calculations resulting from inaccurate date of injury, date of admission, date of treatment, and date of discharge in the KATH orthopaedic trauma registry, Kumasi, Ghana, 2017–2018. Time periods were considered implausible if <0 or ≥90 days

Time period, No. (%)	<0 days	≥90 days	Total implausible values
**Days from injury to admission**	30 (3.1)	8 (0.8)	38 (4.0)
**Days from admission to** **treatment**	15 (1.6)	14 (1.5)	29 (3.0)
**Length of stay**	35 (3.6)	30 (3.1)	65 (6.8)

**Figure 1:**
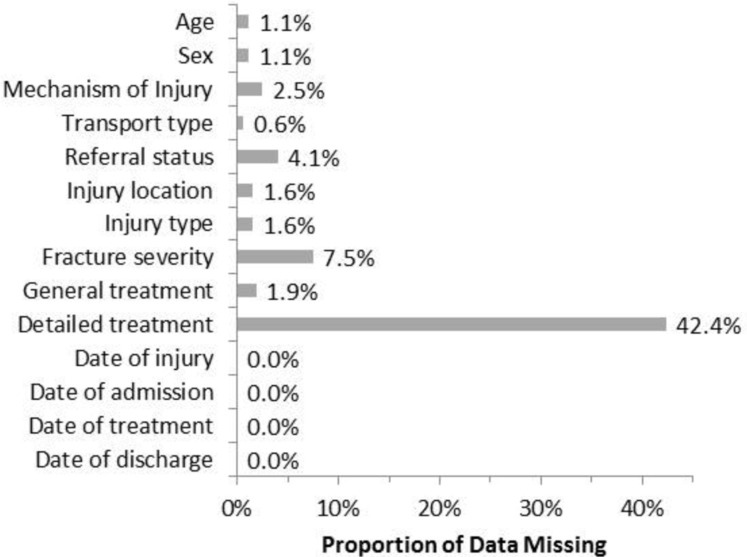
Proportion of data missing on 14 key variables in the KATH orthopaedic trauma registry, Kumasi, Ghana, 2017–2018

In a set of 14 key variables that applied to all patients, missingness was <5% for all but two variables (Figure 1). Detailed treatment was the most frequently missing variable (42.4%), followed by fracture severity (7.5%). Detailed treatment was missing for 73.2% of operative cases and only 0.4% of non-operative cases.

### Key performance indicators

Of 183 femur fractures, 55.2% underwent operative management (n=101). An additional 30.1% were placed in traction without definitive operative management documented (n=55). Of 220 open fractures, 64.1% underwent operative management (n=141), and 64.3% of operations were within 24 hours of admission (133/141).

### Utility of the Orthopaedic Trauma Registry

This description of the utility of the KATH orthopaedic trauma registry is based on the opinions of the three co-authors of this article who are active in-patient care at KATH. Since the start of this registry, the data have been used to successfully advocate for more human resources, operating theatre space, equipment, and supplies. The numbers of admissions and operations were presented to KATH hospital administration to successfully advocate for more medical officers for the orthopaedics department, and more orthopaedic equipment, including power drills and operating room tables, given the increasing number of orthopaedic trauma patients. The data from this registry were useful for this purpose as numbers of operations, and even the number of admissions for fractures, are not well documented in the hospital's medical record department.

The orthopaedic trauma registry also identified several fracture complications among patients who received initial traditional treatment of simple fractures because they could not afford hospital-based treatment. KATH surgeons used these data to secure fracture treatment materials from benevolent organisations for trauma patients who cannot afford materials to treat their fractures.

Finally, the existence of the orthopaedic trauma registry has helped to stimulate the otherwise minimal quality improvement activities at KATH. This has allowed us to identify opportunities for quality improvement within the department. This includes issues directly related to data in this registry, such as initial splinting and bandaging of long bone fractures in the ED.

It also includes issues indirectly related to this registry, through the increased attention to quality improvement that it stimulated, including recognition and resuscitation of patients arriving with hemorrhagic shock, use of cervical collars, and staffing in the ED.

## Discussion

In this evaluation of the KATH orthopaedic trauma registry, we examined patient and injury characteristics of included individuals, experiences in data collection and management, the quality of the data, and the utility of this registry. We found that road traffic injuries were the predominant mechanism of injury leading to orthopaedic injuries and that lower extremity fractures were the most common type of injury. The majority of injuries were managed non-operatively. Overall, the majority of data were internally consistent, but there were some inconsistencies with dates of injury and treatment. Most variables had a low level of missingness, except the type of operative treatment. Despite the difficulties in data gathering and the limitations in the data, the orthopaedic trauma registry has successfully been used to advocate for more resources to care for these injured patients adequately.

Our findings are consistent with the existing literature on trauma registries in LMICs. Road traffic collisions account for a large portion of both orthopaedic and nonorthopaedic injuries. 22, 24, 27, 28 Young and middle-aged adult males are the largest affected demographic group. Similar to orthopaedic injuries at Aga Khan University Hospital in Pakistan, lower extremity fractures were the most common type of injury; however, 90% of injuries in Pakistan were managed operatively compared to only 44% in our study.^23^ The orthopaedic trauma registry in Uganda reported that 43% of injuries were of the lower extremity compared to 60% in our study. The Uganda registry did not report on operative management.^24^ The distribution of type of injury differed in our study compared to injuries seen in Victoria, Australia.

While the majority of injuries were still due to road traffic collisions, spine fractures accounted for 24% of injuries, and only 25% of injuries were in the lower extremity. While the majority of lower extremity fractures were managed operatively, spine fractures were mostly treated non-operatively.^12^

Many trauma registries, particularly in LMICs, report issues with data collection, data quality and missingness.^29^ In the development of the orthopaedic trauma registry at Aga Khan University in Pakistan, authors, report lack of funds for registry software, lack of human resources for night and weekend hours, and lack of proper documentation in the pre-hospital and hospital records as barriers to accurate data collection.^23^ We had similar barriers, including incomplete documentation from referring facilities and in the ED. Our research assistants were assigned weekends and public holidays, patients were not missed at these times. We performed internal consistency checks on dates included in the registry. Nearly 10% of entries had inconsistencies in dates leading to inaccurate calculations in time to seeking care, time to operative management, and hospital length of stay. In validation of a trauma registry at a level II trauma center in the United States, authors reported an error rate of 1% for implausible dates, which was achieved using specific registry software that detected implausible dates in real-time, allowing for immediate correction by the data entry team. 30 The initial database used at KATH did not allow for immediate detection of implausible dates; however, we have since changed to REDCap electronic data capture tool, which allows for more checks on internal consistency.

In terms of data completeness, several trauma registries have reported a goal of at least 80% data completeness.^31^, ^32^ We achieved >90% completeness on all collected variables except details of treatment, particularly type of operative management. Details of operative treatment were frequently missing because many patients were discharged home from ED with instructions to follow up as an outpatient to schedule operative management. Some patients did not follow up or left the hospital against medical advice, but we also had difficulty ensuring completion of data collection once the patient represented for definitive operative care.

### Improvements in the KATH Orthopaedic Trauma Registry

We have made several steps to improve the utility of the KATH orthopaedic registry. In September 2017, we expanded the age range to include children and in August 2019, we added outcome data including: death, discharge against medical advice, implant infection, implant failure, and reoperation.

In December 2019, we converted the database to REDCap, which allows for better standardisation of data entry.

We created a standardised codebook of categorical variables such as mechanism of injury, occupation, diagnosis, and treatment. We added more fields to allow for one diagnosis and one treatment per field, to accurately describe which treatment pertains to each injury, if a patient has multiple injuries. Future directions for the registry include adding variables on non-orthopaedic injuries and management, overall injury severity scoring, and specific orthopaedic injury severity scoring (e.g. fracture complexity). We also plan to enact quality control measures to improve internal consistency, including weekly review of entries to identify missing data and flagging incompletely entered or inconsistent data.

### Limitations of the Orthopaedic Trauma Registry and of this evaluation

This article has several limitations. While one purpose of this evaluation was to examine patient and injury characteristics of individuals included in the orthopaedic trauma registry, we found a relatively high level of data errors based on internal consistency and had a high level of missingness on type of operative management. Issues with data quality and completeness call into question the accuracy of some of the data, particularly injury management. We reported on process of care measures such as percent of femur fractures managed with operative fixation; however, the accuracy of this data is unclear.

In assessing data quality, we were unable to assess the level of data capture. We do not know what per cent of patients eligible for the orthopaedic trauma registry was included in this registry. Also, we were only able to assess the internal accuracy of the data by looking for conflicting values across available variables. Due to funding and human resource constraints, we could not validate the orthopaedic trauma registry data compared to patient charts. We also did not assess inter-rater reliability across data collectors.

## Conclusion

We describe the start of a speciality-specific orthopaedic trauma registry in a lower-middle-income country, along with an assessment of the quality of the data captured. We demonstrate that it is possible to start an orthopaedic trauma registry in this environment with minimal resource requirements. There were major challenges of data consistency and completeness. Despite these limitations, a low-cost specialty-specific registry can be used to advocate for increased resources and to identify areas for quality improvement. By assessing the quality of registry data, we were able to identify several steps to take to improve the registry. It should also be noted that this registry is administered and funded internally by KATH. It is an ongoing patient management tool and not a one-time study. Hence, the above-noted issues with missingness and data quality must be put into perspective of the sustainability and documented utility of the registry.
